# Design an Epoxy Coating with TiO_2_/GO/PANI Nanocomposites for Enhancing Corrosion Resistance of Q235 Carbon Steel

**DOI:** 10.3390/ma14102629

**Published:** 2021-05-18

**Authors:** Shimin Chen, Bo Li, Rengui Xiao, Huanhu Luo, Siwu Yu, Jinghang He, Xia Liao

**Affiliations:** 1 School of Chemistry and Chemical Engineering, Guizhou University, Guiyang 550025, China; smchen0416@163.com (S.C.); tigerlhh@sina.com (H.L.); xliao@gzu.edu.cn (X.L.); 2 Electric Power Research Institute of Guizhou Power Grid Co., Guiyang 550002, China; lbpsylry@163.com (B.L.); swyu2012@163.com (S.Y.); hjh_avitus@126.com (J.H.)

**Keywords:** TiO_2_/GO/PANI nanocomposite, epoxy resin, barrier protection, photocathode protection

## Abstract

In this work, a ternary TiO_2_/Graphene oxide/Polyaniline (TiO_2_/GO/PANI) nanocomposite was synthesized by in situ oxidation and use as a filler on epoxy resin (TiO_2_/GO/PANI/EP), a bifunctional in situ protective coating has been developed and reinforced the Q235 carbon steel protection against corrosion. The structure and optical properties of the obtained composites are characterized by XRD, FTIR, and UV–vis. Compared to bare TiO_2_ and bare Q235, the TiO_2_/GO/PANI/EP coating exhibited prominent photoelectrochemical properties, such as the photocurrent density increased 0.06 A/cm^2^ and the corrosion potential shifted from −651 mV to −851 mV, respectively. The results show that the TiO_2_/GO/PANI nanocomposite has an extended light absorption range and the effective separation of electron-hole pairs improves the photoelectrochemical performance, and also provides cathodic protection to Q235 steel under dark conditions. The TiO_2_/GO/PANI/EP coating can isolate the Q235 steel from the external corrosive environment, and may generally be regarded a useful protective barrier coating to metallic materials. When the TiO_2_/GO/PANI composite is dispersed in the EP, the compactness of the coating is improved and the protective barrier effect is enhanced.

## 1. Introduction

Throughout the development of human civilization, the use of metallic materials has become more and more widespread. However, due to the corrosion of metal materials, huge economic losses and environmental damages have been caused to society. Therefore, effective measures must be taken to prevent corrosion. At present, the most commonly used methods to improve corrosion resistance include protective coatings [1,2,3], corrosion inhibitors [4,5], nanocomposites [6,7], and electrochemical protection [8,9,10,11,12,13]. Electrochemical protection methods mainly include impressed current cathodic protection [8,10], sacrificial anode cathodic protection (SACP) [9,11], and photocathodic protection (PCP) [12,13].

Among various anti-corrosion strategies, coating the metal surface is the most economic, effective and most commonly used method. The coating can form an outer layer that acts as a protective surface barrier to prevent the penetration of external corrosive substances including water, oxygen, or ions. Among various coatings, polymers have special advantages as barrier materials. Epoxy resin (EP) has been widely used as a general-purpose material to improve corrosion resistances due to its excellent chemical resistance and adhesion, good wear resistance and its flexibility. However, since the EP curing process is prone to coating defects such as holes, water channels, and gaps [14,15], corrosive particles may penetrate the coating and cause serious metal corrosion [16]. Adding conductive polymers and nano-fillers to the epoxy resin matrix can not only improve the electrostatic conductivity of the coating, but also promote the formation of a passivation layer at the metal/polymer interface, which can effectively prevent the penetration of corrosive particles. W.F. Huang [17] et al. designed and fabricated a new type of super-hydrophobic polyaniline (PANI)–TiO_2_ based layered nano-composite coating employing a surfactant-free nano-precipitation method. Their results show that the (PANI)–TiO_2_ nano-composite coating is generally comparable to traditional epoxy coatings, but exhibits superior water resistance (water contact angle > 150°) and better anti-corrosion performance.

PCP is a new type of electrochemical anti-corrosion technology, which has the advantages that it is environmentally friendly and energy efficient. A semiconducting film is used as a photoanode, which, under light illumination, can photo-generate electron and hole pairs due to the potential difference between the photoanode and the metal. The photo-generated electrons are transferred to the metal surface to provide cathodic protection. For this purpose, anatase nano-TiO_2_ may be regarded an excellent photocatalytic semiconducting material. Since the inherent band gap of TiO_2_ is 3.2 eV, it can only absorb ultraviolet light, and the photo-generated electrons and holes generated easily recombine [18], limiting the use of TiO_2_. At present, the main methods to modify the photoelectrochemical performance of TiO_2_ include metal ion or non-metal ion doping [19,20], coupling with narrow band gap semiconducting materials [21,22], precious metal deposition [23,24], nanostructure construction [25], and polymer modification [26]. Graphene oxide (GO) is the oxide of graphene (GR), where the layered structure of GR remains after oxidation. At the same time, many oxygen-containing functional groups are introduced into each layer of monolithic GO. Therefore, in addition to some of the basic properties of GR itself, such as large specific surface area [27], and excellent electrical and thermal conductivity [28], GO also has many particular characteristics, such as a good dispersion capability. By the combination of GO and TiO_2_, the higher charge mobility and lamellar structure of GO can increase the photo-generated charge transfer efficiency and reduce the consumption of free electrons. On the other hand, it can slow down the damage to the coating by corrosive particles such as oxygen and water. PANI is considered to be one of the most promising conductive polymers [29], with excellent electrochemical performance and visible light absorption capacity, as well as certain energy storage and physical shielding functionality [30]. Doping PANI can not only effectively transfer holes and promote the separation of photogenerated carriers, but also broaden the light response range of the composite material and improve its utilization rate of the sunlight [31].

Xia [32] et al. produced RGO/TiO_2_ composites and used them as fillers for water-based polyacrylate (PA) coatings. Their results show that the RGO/TiO_2_–PA coating is an excellent barrier layer, which can prevent corrosion in the dark and provide photocathode protection under light. Zhang [33] et al. synthesized a ternary PANI-graphene-TiO_2_ composite material and spread it evenly on the surface of 304 stainless steel to prevent corrosion. Their results show that under visible light irradiation, the ternary composite material has a stronger light response than pure TiO_2_, and its photoelectrochemical performance is significantly enhanced. Li [34] et al. fabricated CdTe/graphene/TiO_2_ films by an electrochemical deposition method, where their results show that CdTe/graphene/TiO_2_ composite materials exhibit high light absorption in both ultraviolet and visible light regions, and offer a good photocathode protection performance. Although many studies can be found in the literature concerning the use of ternary TiO_2_ hybrids as photoanodes for PCP, it generally requires two corrosion cells and photoanode cells connected by wires. This complex equipment seriously affects its practical application [35,36]. On the contrary, there are few studies available on directly using semiconducting materials for in situ protection of metals. Therefore, a simple in situ photocathode protection system is of great significance to the promotion of photocathode protection technology.

**Figure 1:**
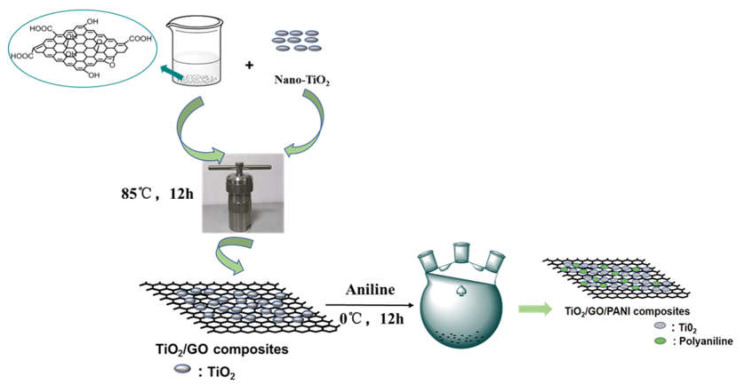
Schematic diagram of the preparation process of TiO_2_/GO/PANI composite.

To further improve anti-corrosion performances, a composite coating protection method is proposed in the present study that combines the traditional method with the photogenerated. TiO_2_/GO, TiO_2_/PANI, and TiO_2_/GO/PANI composite materials were gradually synthesized by a one-step hydrothermal method and in situ oxidative polymerization. As shown as Figure 1, the GO dispersion, and nano-TiO_2_ transferring to a reaction kettle operated at 85 °C for 12 h of hydrothermal reaction followed. After centrifugation, drying and grinding, the TiO_2_/GO nanocomposite was synthesized. Then, the TiO_2_/GO nanocomposite was added to the aniline-containing emulsion for in situ polymerization. Finally, the TiO_2_/GO/PANI composite material was used as a filler with EP as the film-forming substrate to form a composite protective coating, which was directly deposited onto Q235 carbon steel plates to evaluate the photocathode protection effect and anti-corrosion performance. Results show the addition of TiO_2_/GO/PANI nanocomposite can greatly improve the anticorrosion performance of EP-coating. Hypothesized mechanisms for the corrosion resistance of the TiO_2_/GO/PANI/EP was discussed. The composites provide improved photocathodic protection under visible light illumination and physical barrier layer protection in dark condition.

## 2. Materials and Methods

### 2.1. Materials

TiO_2_ nanoparticles (99.8% metals basis, 5–10 nm, anatase) was obtained from Shanghai Macklin Biochemical Technology Co., Ltd. (Shanghai, China). Natural graphite flakes (1200 mesh) was obtained from Shenzhen Hanhui Graphite Co., Ltd. (Shenzhen, China). Aniline was freshly distilled before use, which purchased from Aladdin. H_2_SO_4_ (98.3%), HCl (36–38%) and hydrogen peroxide (H_2_O_2_, 30%) were obtained from Chongqing Chuangdong Chemical (group) Co., Ltd. (Chongqing, China). Potassium permanganate (KMnO_4_) and ammonium persulfate (APS) were bought from Chengdu Jinshan Chemical Reagent Co., Ltd. (Chengdu, China). Sodium nitrate (NaNO_3_), 1-Methyl-2-pyrrolidinone (NMP) and n-butanol were all purchased from Tianjin Kemiou Chemical Reagent Co., Ltd. (Tianjin, China). Epoxy resin (E-44) and the corresponding curing agent (T-31) was kindly supplied by Changzhou Runxiang Chemical Co., Ltd. (Changzhou, China), respectively. The mass ratio of E-44 and T-31 is 2:1.

### 2.2. Preparation of Binary TiO_2_/GO Composite Materials

The modified Hummers method was used to prepare graphene oxide, and the one-step hydrothermal method was used to prepare binary TiO_2_/GO composites. A quantity of 75 mg of layered GO was mixed with 100 mL deionized water in a beaker for ultrasonic dispersion for 2 h. Then, 1 g of TiO_2_ was added and ultrasonically dispersed for 1 h, followed by transferring the resulting suspension to a reaction kettle operated at 85 °C for 12 h of hydrothermal reaction, followed by centrifugation. After drying and grinding, a binary TiO_2_/GO composite material was obtained.

### 2.3. Preparation of Binary TiO_2_/PANI Composite Material

Binary TiO_2_/PANI composite material was synthesized by in situ polymerization. A quantity of 1 g of TiO_2_ was mixed with 60 mL of 0.6 mol/L dilute HCl in a three-necked flask, and was stirred for 30 min to achieve full dispersion. Next, 3 mL of Aniline were added under constant stirring for 30 min. Additionally, a 60 mL 0.6 mol/L dilute HCl solution was prepared, and 9.15 g of APS were added. Next, the APS-containing solution was slowly added to the three-necked flask to continue the polymerization at 0 °C for 24 h. The resulting product was washed with absolute ethanol and deionized water to neutrality, followed by drying at 60 °C for 12 h and grinding. The final product was identified as the desired binary TiO_2_/PANI composite material.

### 2.4. Preparation of Ternary TiO_2_/GO/PANI Composite Material

The ternary TiO_2_/GO/PANI composite material was also synthesized by in situ polymerization. A quantity of 1 g of TiO_2_ was mixed with 60 mL of 0.6 mol/L dilute HCl in a three-necked flask under constant stirring for 30 min. 60 mL of dispersed graphene oxide was added under continued stirring for 30 min to achieve full dispersion. Furthermore, 3 g of Aniline were added under constant stirring for 30 min. Additionally, a solution of 60 mL 0.6 mol/L dilute HCl was prepared and 9.15 g of APS were added. Next, the APS containing solution was slowly added to the three-neck flask to continue the polymerization at 0 °C for 24 h. The final product was washed with absolute ethanol and deionized water to neutrality, followed by drying at 60 °C for 12 h. The final product was identified as the desired ternary TiO_2_/GO/PANI composite material.

### 2.5. Preparation of Quaternary TiO_2_/GO/PANI/EP Composite Coating

A quantity of 1 g of the TiO_2_/GO/PANI composite material was mixed with 7.28 g of NMP, 2.72 g of n-butanol, and 20 g of E-44. After blending and stirring at high speed for 2 h, 10 g of T-31 were added and the mixture was further stirred for 30 min. The resulting TiO_2_/GO/PANI/EP composite coating onto the surface of Q235 carbon steel that had been polished, cleaned and cured at room temperature for 24 h.

### 2.6. Characterizations

A Nicolet-iS5 Fourier Transform Infrared Spectrometer (FT-IR) from Thermo Fisher Scientific (Waltham, MA, USA) was used to analyze the functional group composition of the synthesized composite material. Potassium bromide was used as the calibration substance over a wave number range of 500–4000 cm^−1^. X’PERT X-ray diffraction (XRD) was performed using of PANalytical BV diffractometer (Almelo, The Netherlands) to analyze the crystal structure of the synthesized composite material. The tube voltage and current were 40 KV and 40 mV, respectively. Raman was carried out on WITec alpha300R confocal Raman microscopy (WITec GmbH, Ulm, Germany). The excitation laser is a diode-pumped solid-state laser (532 nm, cobalt Laser). A SIGMA field emission scanning electron microscope (SEM) from Zeiss (Oberkochen, Germany) was used to study the morphology of the composite coating. The Particle size distribution of the GO after sonication was carried out on Beckman Coulter LS13320MW. A U4100 Ultraviolet Visible Diffuse Reflectance Spectrometer (UV-DRS) from Hitachi (Tokyo, Japan) was used to measure the light absorption properties of the composite materials with BaSO_4_ reference using a scanning range of 200–800 nm. A CHI760E electrochemical workstation from Shanghai ChenHua (Shanghai, China) was used to characterize the photoelectrochemical performance of the samples. The electrochemical analysis was performed with a three-electrode system, using saturated calomel as the reference electrode, platinum sheet as the counter electrode, Q235 as the working electrode, and 3.5% NaCl as the electrolyte. The light source was a 300 W xenon lamp (PLS-SXE300UV, Beijing Perfect Light Co. Ltd., Beijing, China) with an AM1.5 (simulated sunlight). The photocurrent density with time (I-T) curves and OCP-T were tested at a 0 V (Hg/HgO) bias potential. Electrochemical impedance spectroscopy (EIS) was performed over the frequency range of 10^5^ to 10^−2^ Hz with an amplitude of 5 mV. and the potentiodynamic polarization curves were plotted with 10 mV/s scan rate.

## 3. Results

### 3.1. Structure Characterization

**Figure 2:**
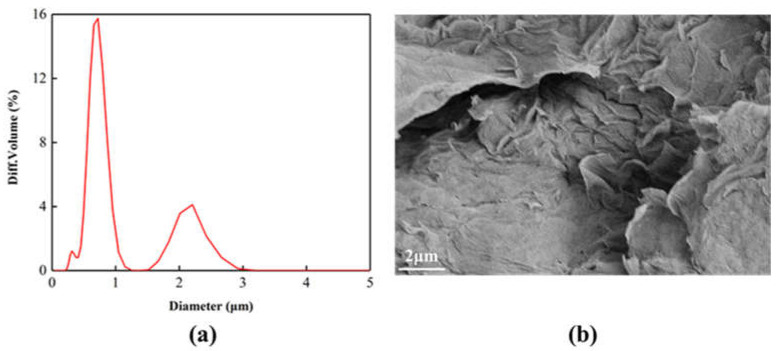
(**a**) Particle size distribution, (**b**) SEM images of the GO after sonication.

The Particle size distribution of the GO after sonication is shown in Figure 2a. The GO particle size is concentrated at 0.7 μm. Figure 2b is the SEM image of GO. It can be seen that the surface of GO is relatively smooth, showing accumulation phenomenon, and the surface is slightly wrinkled. This is because the surface of GO contains a large number of oxygen-containing functional groups. There is a strong interaction force between them, which causes the layers to pile up on each other.

**Figure 3:**
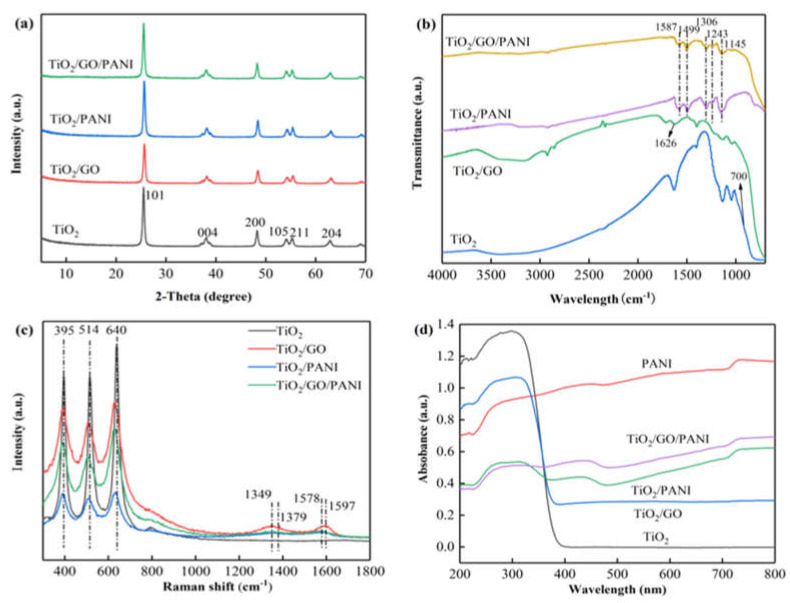
(**a**) XRD patterns, (**b**) FTIR spectra, (**c**) Raman spectra, (**d**) UV–vis DRS.

The XRD patterns of the TiO_2_, TiO_2_/GO, TiO_2_/PANI, and TiO_2_/GO/PANI nanocomposites are presented in Figure 3a Diffraction peaks are found at 2 θ values of 25.5°, 37.9°, 48°, 54.2°, 55.3°, and 62.7°, which correspond to the (101), (004), (200), (105), (211), and (204) reflections of the tetragonal TiO_2_ anatase phase [37]. After the introduction of GO and PANI, no new characteristic peaks appeared in the composite material, and the TiO_2_ reflections are unaffected. This implies that the addition of GO and PANI had no effect on the crystallization behavior of TiO_2_. The average grain size of the TiO_2_ particles in the TiO_2_/GO/PANI composite material was calculated to be 20 nm using the Debye–Scherrer formula.

The FTIR spectra of the synthesized TiO_2_, TiO_2_/GO, TiO_2_/PANI, and TiO_2_/GO/PANI composites are shown in Figure 3b. It can be seen that the O–H stretching vibration peak at 3400 cm^−1^ is reduced when TiO_2_ is mixed with GO, and it even disappears when the PANI is added, which proves that TiO_2_ interacts with GO and PANI through surface chemical reactions. In the TiO_2_/GO spectrum, the characteristic peaks of the partial oxygen-containing groups in GO are weakened, indicating that GO is partially reduced under hydrothermal conditions. The absorption peak at 1626 cm^−1^ is the C=C stretching vibration mode in GO. The SP^2^ hybrid orbital of GO [38] is clearly detectable, proving the existence of GO. In the TiO_2_/PANI and TiO_2_/GO/PANI spectra the C=C stretching vibration modes of the quinone and benzene rings are displayed at around 1587 cm^−1^ and 1599 cm^−1^ [39], respectively, whereas the modes at around 1312 cm^−1^ and 1243 cm^−1^ correspond to the C–N stretching vibration in the quinone ring and benzene ring, respectively. The strong absorption band at 1145 cm^−1^ in the TiO_2_/PANI and TiO_2_/GO/PANI spectra is a characteristic feature of the PANI conductivity manifesting electron delocalization [40], which proves the existence of PANI. The flat peak at around 700 cm^−1^ is visible in all the spectra and corresponds to the vibration peak of Ti–O–Ti and Ti–O–C, which is the characteristic absorption band of TiO_2_ [41].

The Raman spectra of the prepared samples (Figure 3c) can further support the formation of the ternary TiO_2_/GO/PANI nanocomposites. The two strong peaks at 1348 and 1597 cm^−1^, namely, D and G peaks of GO can be found in the GO-involved samples. The G peak is caused by the stretching vibration mode of all sp^2^ atom pairs in the carbon ring or long-chain, while the D peak is caused by the breathing vibration mode of the sp^2^ atom in the carbon ring. The appearance of the D peak indicates that there is a disordered state in the graphite lattice, indicating that after the graphite is oxidized, a part of the sp^2^ hybridized carbon atoms in the structure is transformed into an sp^3^ hybrid structure; that is, the C=C double bond in the graphite layer is destroyed. Characteristic peaks of PANI are found at around 1379 and 1568 cm^−1^, which can be ascribed to protonated C–N stretching and C–C stretching in the benzene or quinoid ring, respectively. The bands at about 395, 515, and 640 cm^−1^ belong to TiO_2_ nanoparticles.

Figure 3d displays the UV-DRS of the TiO_2_, TiO_2_/GO, TiO_2_/PANI, and TiO_2_/GO/PANI composites. For nanoparticles used in photoelectrochemical materials such as TiO_2_ based photoelectrochemical anticorrosion coatings, the relative light wave response range is an important parameter. It can be seen from the figure that pure TiO_2_ shows an absorption edge at about 380 nm due to its inherent band gap absorption. After doping with GO and PANI, the absorption intensity of the TiO_2_/GO, TiO_2_/PANI, and TiO_2_/GO/PANI nanocomposites in the ultraviolet and visible light region is significantly improved, and the absorption edge is redshifted. This red shift phenomenon may be due to the electronic interaction between GO and TiO_2_ semiconductor materials, forming Ti–O–Ti and Ti–O–C bonds, which then results in the expansion of the light absorption range [42]. Furthermore, the π-π* electronic transition of the benzene ring structure in the PANI molecule and the n-π* polaron transition of the benzene and the quinone rings leads to higher absorption in the range of ultraviolet and visible light [43]. This implies that the prepared composite materials broaden the light wave response range and the absorption intensity. This is an important feature to help improving the utilization of solar energy.

### 3.2. Photoelectrochemical Performance

The photoelectric–chemical conversion of TiO_2_/GO/PANI/EP is very important. Thus, photoelectric–chemical conversion performance characterization was carried out by testing photocurrent and open circuit potential of working electrode coated with TiO_2_/GO/PANI/EP at same light intensity.

**Figure 4:**
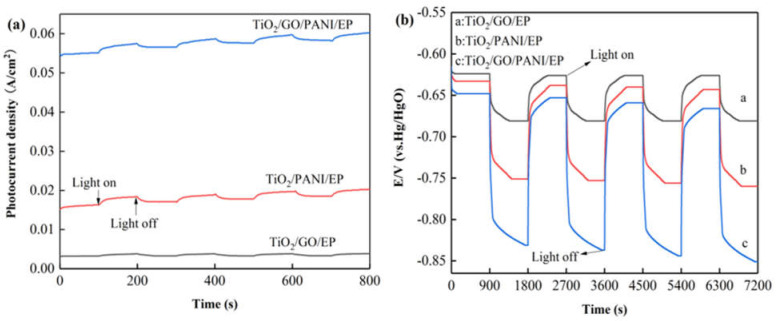
(**a**) I-t curve (vs. Hg/HgO) (**b**) OCP-t curves (vs. Hg/HgO) of the Q235 coated with TiO_2/_GO/EP, TiO_2_/PANI/EP, and TiO_2_/GO/PANI/EP coating under intermittent light on and off.

Figure 4a shows the curve of the photocurrent density as a function of irradiation time of the Q235 coated with TiO_2_/GO/EP, TiO_2_/PANI/EP and TiO_2_/GO/PANI/EP coating under intermittent light on and off. It can be seen from the photocurrent diagram that during 100 s illumination the photocurrent the photocurrent is relatively stable at a positive value, indicating that most of the photogenerated electrons and holes can be separated before recombination. Electrons are in situ transferred to the coupled metal and, thereby, achieve cathodic protection. All the samples showed an instantaneous photocurrent response when the light was turned on, and the photocurrent gradually disappeared after turning off the light. However, the electrolyte may diffuse into the coating [44], and thus the photocurrent density did not return to its original value after each switch off, leading to a gradual increase of the photocurrent curve. and the photocurrent response did not change, indicating that the coating has excellent stability, which is essential for long-term practical applications. In addition, it can be observed that the photocurrent density of TiO_2_/GO/PANI/EP is the highest, which can reach up to 0.06 A/cm^2^. This result may be explained by a synergetic effect of the broadening of the spectral absorption range and the improvement of the separation efficiency of photo-generated electron-holes. The extremely high electron transfer efficiency of GO on the one hand reduces the recombination efficiency of photogenerated electron-hole pairs, while on the other hand it can change the migration direction of photogenerated electrons. Furthermore, the PANI may also expand the light absorption range and promote the generation of photoelectrons and their separation from the holes.

Figure 4b displays shows the changes of the open circuit potential (OCP) curves for the Q235 coated with TiO_2_/GO/EP, TiO_2_/PANI/EP, and TiO_2_/GO/PANI/EP coating under intermittent light on and off. From the OCP curves it can be seen that after Q235 is coupled with the coating in situ, the light irradiation causes the composite coating to continuously generate photo-electrons, which migrate to the Q235 surface, which in turn reduces the corrosion potential and provides cathodic protection [45]. The corrosion potential of the TiO_2_/GO/EP composite coating is reduced from −631 to −681 mV, whereas the corrosion potential of the TiO_2_/PANI/EP composite coating is reduced from −633 to −760 mV, and the corrosion potential of the TiO_2_/GO/PANI/EP composite coating is reduced from −651 to −851 mV. These results show that the TiO_2_/GO/PANI/EP composite coating has the largest change in corrosion potential and the lowest total corrosion potential, which implies that TiO_2_/GO/PANI/EP exhibits the best cathodic protection performance as compared with the other coatings. In addition, the OCP negative shift value of the TiO_2_/GO/EP, TiO_2_/PANI/EP, and TiO_2_/GO/PANI/EP composite coatings can still reach the initial level after the lamp is repeatedly switched on and off, indicating that the coating has good stability and repeatability. On the other hand, the potential of the TiO_2_/PANI/EP and TiO_2_/GO/PANI/EP coatings did not return to the initial value, but became more and more negative. Even after the light was turned off, the potential remained below the self-corrosion potential of the in situ coupled Q235 carbon steel for a certain period of time. This indicates that the coating provides a certain degree of cathodic protection even after the light is turned off. This phenomenon may be caused by the charge and discharge behavior of the photocatalytic system before and after light illumination. PANI is a pseudo-capacitance electrode material with a high capacitance and good energy storage functionality. Therefore, the prepared composite coating has the possibility of providing cathodic protection to Q235 also under dark conditions.

### 3.3. Morphology of Coatings

**Figure 5:**
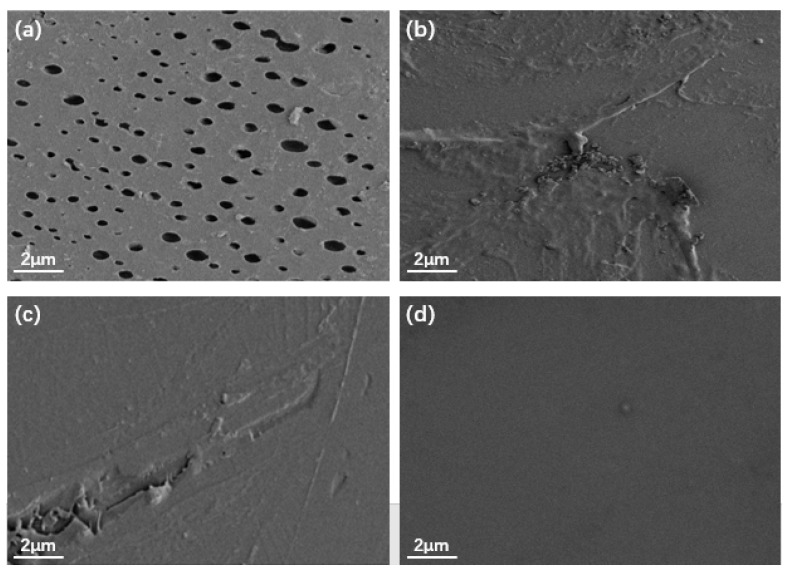
SEM images of (**a**) EP, (**b**) TiO_2/_GO/EP, (**c**) TiO_2_/PANI/EP, (**d**) TiO_2_/GO/PANI/EP.

Figure 5 shows the SEM images of the EP, TiO_2_/GO/EP, TiO_2_/PANI/EP, and TiO_2_/GO/PANI/EP coatings. It can be seen from the Figure 5a that there are many micropores on the surface of the EP coating, whereas the surfaces of the coatings depicted in Figure 5b–d are relatively dense, without any obvious pores. This indicates that the nanocomposite particles added to the EP coating can effectively fill the micropores and defects in the EP coating. However, the surface of the Figure 5b,c coatings is relatively rough, with a certain degree of agglomeration and protrusions, while the surface of the Figure 5d coating is relatively smooth, it shows that the ternary TiO_2_/GO/PANI nanocomposite has the best dispersibility in EP.

### 3.4. Corrosion Resistance Property of Coatings

**Figure 6:**
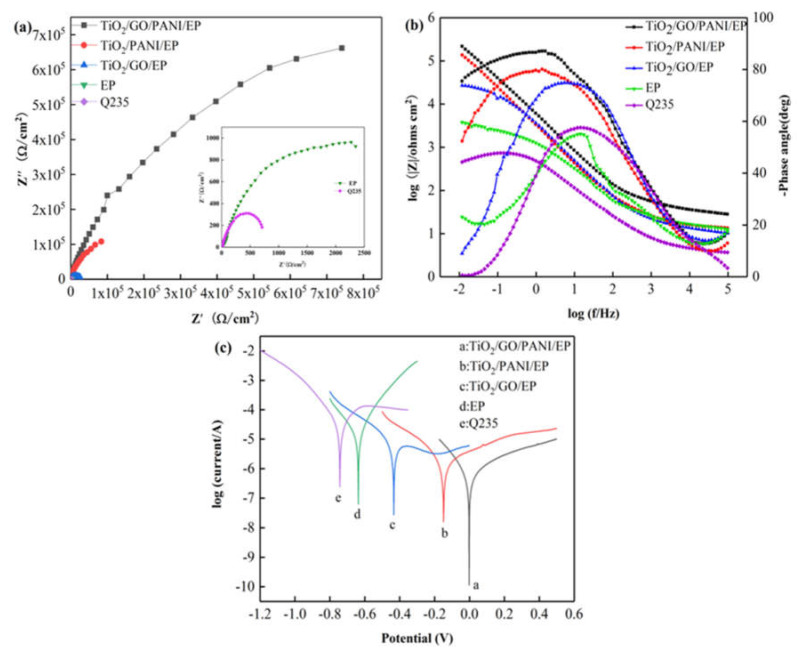
The EIS and Tafel curves for the EP, TiO_2_/GO/EP, TiO_2_/PANI/EP, and TiO_2_/GO/PANI/EP composites coated Q235 in the dark in 3.5 wt % NaCl aqueous solution under light off (**a**) Nyquist plot, (**b**) Bode and Phase plot, (**c**) Polarization curves.

Figure 6a,b show the Nyquist and Bode diagrams, respectively, of the Q235 carbon steel, EP, TiO_2_/GO/EP, TiO_2_/PANI/EP, and TiO_2_/GO/PANI/EP composite coatings in 3.5% NaCl in the dark state. Over the entire frequency range, the Nyquist plots all show approximate semicircles with different diameters. The size of the capacitive arc directly reflects the resistance of the corrosive particles when passing through the composite coating. The larger the radius of the capacitive arc, the longer the time required for the electrolyte solution to corrode the coating and the better the corrosion resistance. It can be seen from the figure that the capacitive reactance arc radius of each sample has the following results: TiO_2_/GO/PANI/EP > TiO_2_/PANI/EP > TiO_2_/GO/EP > EP > Q235. Compared with the capacitive arc radius of bare carbon steel and the EP coating, the arc radius for the TiO_2_/GO/PANI/EP, TiO_2_/PANI/EP, and TiO_2_/GO/EP coatings is larger, indicating that the composite coating has an anti-corrosion effect on the carbon steel substrate. Since the TiO_2_/GO/PANI/EP coating has the largest capacitive arc radius, it offers the best anti-corrosion performance. It can be seen from Figure 6b that the phase angle of the TiO_2_/GO/PANI/EP coating is close to the value of −90° typical for an ideal capacitor over a wide frequency range. This indicates that the coating can resist the penetration of corrosive particles into the surface of the Q235 substrate and the corrosive particles accumulate in the coating as in a charge storage device, i.e., capacitor. The |Z| value has increased by two orders of magnitude compared with the pure EP coating, which may be due to the fact that the TiO_2_/GO/PANI/EP filler can be better dispersed in the EP by filling the defects and preventing the penetration of corrosive particles and, thus, has the best anti-corrosion performance. Figure 6c shows the Tafel polarization curves of the composite coatings. It can be seen that after adding TiO_2_/GO/EP, TiO_2_/PANI/EP, and TiO_2_/GO/PANI/EP fillers to the EP, the corrosion voltage shifts to the right and the corrosion current density is significantly reduced, indicating that the composite fillers added to the EP coating can greatly improve the anti-corrosion performance. Compared with the TiO_2_/GO/EP and TiO_2_/PANI/EP coatings, the TiO_2_/GO/PANI/EP coating displays a higher corrosion voltage and lower corrosion current. This may be due to the good compatibility of the TiO_2_/GO/PANI/EP filler with EP. Under the action of multiple barriers, it prevents the penetration of corrosive particles to the metal surface, which effectively improves the corrosion resistance.

**Figure 7:**
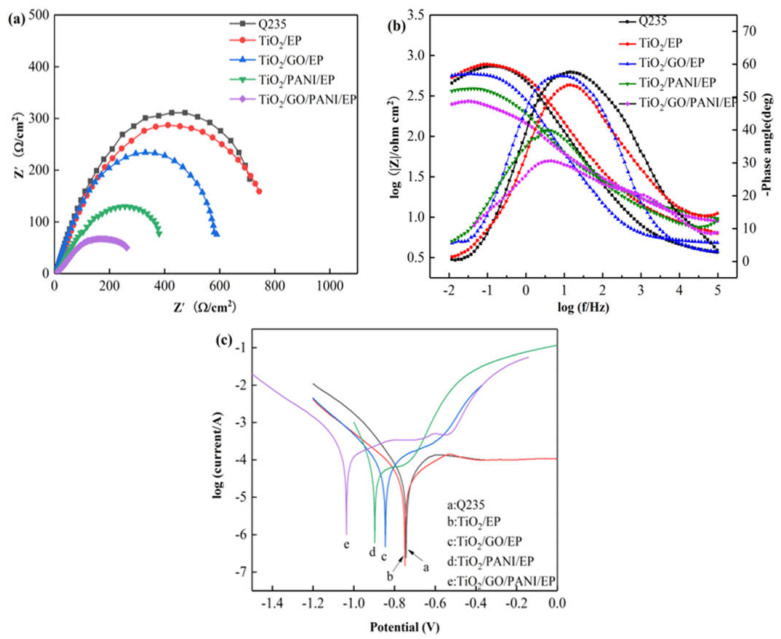
The EIS and Tafel curves for the EP, TiO_2/_GO/EP, TiO_2_/PANI/EP, and TiO_2_/GO/PANI/EP composites coated Q235 under illumination in in 3.5 wt % NaCl aqueous solution under light off (**a**) Nyquist plot, (**b**) Bode and Phase plot, (**c**) Polarization curves.

Figure 7a,b show the equivalent Nyquist and Bode diagrams as in Figure 7 for the Q235 carbon steel, TiO_2_, TiO_2_/GO/EP, TiO_2_/PANI/EP, and TiO_2_/GO/PANI/EP composite coatings in 3.5% NaCl under light illumination. The arc radius of the capacitive reactance directly reflects the electron transfer efficiency of the semiconductor under light. Generally, the smaller the capacitive reactance arc radius of the coating, the higher the conductivity of the coating, and the higher the electron migration rate of the semiconductor. It can be seen from Figure 7a that the capacitive arc radius of all coatings is reduced as compared to the pure TiO_2_ coating, whereby the arc radius of the TiO_2_/GO/PANI/EP coating is the smallest, indicating that the photogenerated electron-hole pair has the fastest transmission speed. The better the conductivity, the better cathodic protection performance. It can be seen from Figure 7b that the phase angle of the TiO_2_/GO/PANI/EP coating is the smallest and the |Z| value is the lowest, which again indicates that the photogenerated electrons are transmitted to the metal surface faster and the photoelectrochemical reaction activity is higher. Figure 7c shows the Tafel polarization curves of the composite coatings. It can be seen that the corrosion potential of all coatings is significantly shifted to the left compared to the Q235 carbon steel, and the corrosion current density is significantly increased. Under light illumination, the semiconductor produces a large number of photogenerated electrons, which transfer to the surface of the Q235 carbon steel substrate, and the corrosion potential is reduced. In this state the coating provides photocathode protection, where the corrosion potential of the TiO_2_/GO/PANI/EP coating is the lowest and the protection effect is the best.

## 4. Discussion

In this work, we fabricated ternary TiO_2_/GO/PANI composite. This new design strategy of the composite materials is that TiO_2_ serves as a fundamental catalyst, and GO and PANI are used as conductive enhancement networks and energy storage materials, respectively. Then, bifunctional in situ composite coatings were prepared by dispersing the as-prepared sample in EP. The coatings were coated onto the Q235 panel to obtain the integrated electrode. It was exciting that the TiO_2_/GO/PANI/EP was more effective in metal protection than other coatings, including the photocurrent density, which increased by 0.06 A/cm^2^, and the corrosion potential shifted from −651 to −851 mV.

**Figure 8:**
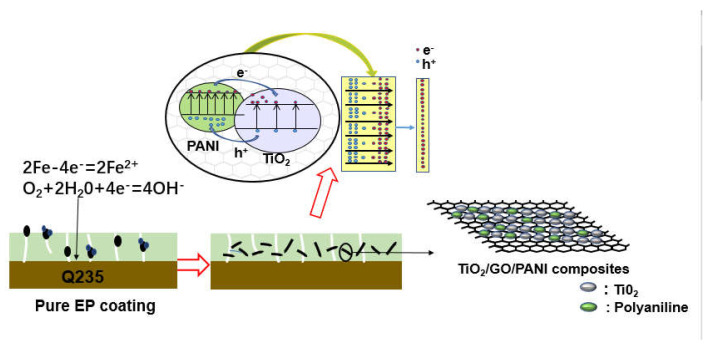
Corrosion resistance mechanism of TiO_2_/GO/PANI/EP coating.

Figure 8 shown the corrosion resistance mechanism of TiO_2_/GO/PANI/EP coating. Generally, an EP coating can isolate and protect the metal from the external corrosive environment. However, during the EP curing process, some micropores will be formed on the metal surface due to the evaporation of the solvent, and the oxidizing medium can easily pass through the pores to reach the metal substrate and cause corrosion. When TiO_2_/GO/PANI is evenly distributed in the EP coating, the composite acts as a filler material, improving the compactness, fills the gaps, and provides an extra barrier layer against corrosive media into the pores and water channels which could prevent steel from corrosion attack. The conductive properties of GO prevent the formation of rust, while evenly dispersed PANI in the coating, exerts a unique anti-corrosion effect that can passivate the metal substrate and delay corrosion. When the TiO_2_/GO/PANI/EP coated Q235 carbon steel is exposed to light, photo-generated electrons and hole pairs are generated. Due to the potential energy difference between the Fermi energy and the excitation energy level, the photogenerated electrons are transferred to the surface of the Q235 carbon steel to shift the corrosion potential in a negative direction. This implies that the TiO_2_/GO/PANI/EP coating provided photo-generated cathodic protection to the Q235 carbon steel. TiO_2_ itself can generate photo-electrons and holes, where the electrons are transported to the metal to prevent the metal to lose electrons, thereby playing a protective role. When the coating contains PANI, photons are absorbed and electron-hole pairs are generated due to its narrow band gap (1.4 eV), which provides free and delocalized electrons and holes. The photo-generated electrons are transferred from the highest occupied molecular orbital (HOMO) to the lowest unoccupied molecular orbital (LUMO) in the conduction band of TiO_2_. The good carrier mobility of GO reduces the recombination of photo-generated electrons and hole pairs, because electrons are transported instantly to the metal to prevent the metal from losing electrons. In this process, since the PANI exhibits high capacitance and charge/discharge performance, it has an energy storage functionality and the TiO_2_/GO/PANI composite material can still release electrons during a certain period of time when the light is turned off, i.e., playing the role of a photocathode protector.

## 5. Conclusions

TiO_2_/GO/PANI nanocomposites were successfully synthesized by in situ oxidative polymerization. Through FT-IR, XRD, UV–Vis DRS, and other methods, the structure, crystal form, and light absorption properties of the material have been studied. The results show that TiO_2_ interacts with GO and PANI through surface chemical reactions; after doping with GO and PANI, the prepared TiO_2_/GO/PANI nanocomposite materials did not appear new characteristic peaks and crystal structures. The addition of GO and PANI did not affect the crystallization behavior of TiO_2_; compared with TiO_2_, the composite material has a significant increase in the absorption intensity of ultraviolet light and visible light, and the absorption edge has a redshift. Efficient separation of photogenerated electrons and holes was indicated, with improved efficiency of electron migration.

The TiO_2_/GO/PANI composite material was used as a filler with EP as the film-forming substrate to form TiO_2_/GO/PANI/EP bifunctional in situ composite coating on the surface of Q235 carbon steel. The TiO_2_/GO/PANI/EP composite coating offers the most comprehensive protection mechanism with a synergy of the physical shielding effect and the photocathode protection effect.

Since PANI exhibits an energy storage functionality due to its high capacitance and charge/discharge performance, the TiO_2_/GO/PANI composite material can still release electrons during a certain period of time after light illumination is turned off, which constitutes a photocathode protection mechanism.

## Data Availability

Data regarding this article is available from the corresponding author upon a reasonable request.
